# Supplementary material for: Causal Relationship between Aging and Anorexia Nervosa: A White-Matter-Microstructure-Mediated Mendelian Randomization Analysis
Source: Biomedicines. 2024 Aug 16;12(8):1874. doi: 10.3390/biomedicines12081874 (PMC11351342; doi:10.3390/biomedicines12081874)
Supplement: Supplementary file 1 [file biomedicines-12-01874-s001.zip › Supplementary Figure/Figure S8/Leaveoneout plot of MR analysis for TL to ubm-b-1990.pdf]

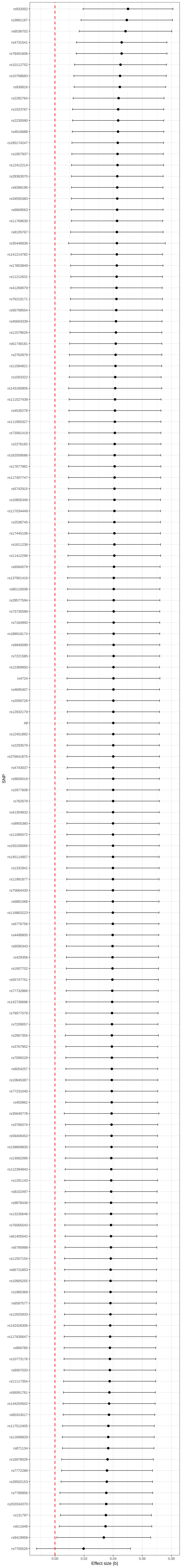
rs932002
rs3891167
rs6536702
rs4731541
rs78491606
rs10112752
rs10768683
rs939916
rs2282764
rs1023767
rs2230590
rs4616688
rs185174247
rs1957937
rs12412214
rs28363070
rs9398196
rs34550383
rs6669563
rs11769630
rs8105767
rs35446936
rs141214782
rs17803849
rs11212631
rs41269079
rs76219171
rs56799554
rs45604339
rs11579626
rs61748181
rs2763979
rs11584821
rs1003322
rs143190905
rs111527438
rs4530278
rs111950327
rs73581419
rs2276182
rs182059586
rs17677991
rs117407747
rs5742915
rs10805346
rs117034449
rs2538745
rs17445108
rs1611236
rs11412296
rs6584579
rs137901416
rs80116508
rs28577594
rs73730598
rs7164950
rs188918174
rs9940099
rs7221585
rs12369950
rs4724
rs4695407
rs2056726
rs12932179
All
rs12451892
rs2293579
rs376641875
rs4743037
rs9600019
rs2977608
rs762679
rs41304832
rs9955360
rs11085072
rs150150565
rs145114957
rs1332941
rs11991877
rs75664430
rs6881568
rs116863223
rs6776756
rs4498805
rs6590343
rs429358
rs1907702
rs55747751
rs77732866
rs142730696
rs79977579
rs7209057
rs2967355
rs3767952
rs7099229
rs6054257
rs10845387
rs77231040
rs450962
rs35640778
rs3785074
rs59409453
rs139669835
rs13062095
rs112394943
rs1291143
rs8102497
rs9878436
rs13230646
rs76065543
rs61405042
rs6790988
rs11557154
rs66731853
rs10905255
rs1985369
rs6587577
rs12925933
rs142426306
rs117630647
rs869785
rs10773176
rs6007020
rs11117354
rs56061761
rs144204502
rs80324517
rs117512405
rs11699829
rs871134
rs16978028
rs7772289
rs28502153
rs7790856
rs202034370
rs131797
rs611646
rs9419958
rs7705526
SNP
0.00
0.02
0.04
0.06
0.08
Effect size (b)
